# Immunogenicity Rates After SARS-CoV-2 Vaccination in People With End-stage Kidney Disease: A Systematic Review and Meta-analysis

**DOI:** 10.1001/jamanetworkopen.2021.31749

**Published:** 2021-10-28

**Authors:** Jia-Jin Chen, Tao Han Lee, Ya-Chung Tian, Cheng-Chia Lee, Pei-Chun Fan, Chih-Hsiang Chang

**Affiliations:** 1 Department of Nephrology, Chang Gung Memorial Hospital, Linkou, Taiwan; 2 Kidney Research Center, Department of Nephrology, Chang Gung Memorial Hospital, Linkou, Taiwan

## Abstract

**Importance:**

Adults receiving dialysis treatment have a higher likelihood of death when infected with SARS-CoV-2 than adults not receiving dialysis treatment. To date, the immune response of people receiving dialysis after SARS-CoV-2 vaccination has not been systematically discussed.

**Objective:**

To assess immunogenicity rates in people with end-stage kidney disease (ESKD) receiving SARS-CoV-2 vaccines, explore postvaccination potential risk factors for nonresponse, and assess whether receiving dialysis is associated with different antibody response rates compared with the nondialysis population.

**Data Sources:**

This systematic review and meta-analysis used articles from PubMed, Medline, and Embase published before July 30, 2021, as well as articles in the *medRxiv* preprint server.

**Study Selection:**

Studies that evaluated the immunogenicity rate according to the postvaccine antibody response rate in patients with ESKD receiving dialysis were selected.

**Data Extraction and Synthesis:**

The meta-analysis was conducted according to the Preferred Reporting Items for Systematic Reviews and Meta-Analyses (PRISMA) guideline. A random-effects model was used. Two independent reviewers conducted the literature search and extracted the data.

**Main Outcomes and Measures:**

The primary outcome was the pooled antibody postvaccine response rates in individuals with ESKD. The secondary outcomes were pooled response rates in individuals receiving and not receiving dialysis. Subgroup analysis and meta-regression were conducted to identify the sources of heterogeneity.

**Results:**

A total of 32 studies were included. The overall immunogenicity rate of the dialysis group was 86% (95% CI, 81%-89%). Meta-regression showed a significant difference was detected in the postvaccine response rate on the basis of prevalence of diabetes (regression coefficient, −0.06; 95% CI, −0.10 to −0.02; *P* = .004). Compared with nondialysis controls, patients in the dialysis group had a lower response rate after the first (relative risk [RR], 0.61; 95% CI, 0.47-0.79; *I*^2^ = 70.2%) and second (RR, 0.88; 95% CI, 0.82-0.93; *I*^2^ = 72.2%) doses, with statistically significantly increased RR between first and second doses (*P* = .007).

**Conclusions and Relevance:**

These findings suggest that the immunogenicity rate among patients receiving dialysis was 41% after the first dose and 89% after the second dose. Diabetes might be a risk factor for nonresponse in the dialysis population. Patients receiving dialysis had a poorer antibody response rate than did individuals not receiving dialysis, particularly after the first dose.

## Key Points

**Question:**

What are the immunogenicity rates in people with end-stage kidney disease receiving SARS-CoV-2 vaccines?

**Findings:**

This systematic review and meta-analysis of 32 studies found that patients receiving dialysis had lower immunogenicity rates after first and second vaccine doses than those not receiving dialysis. Prevalence of diabetes had an inverse linear association with immunogenicity rate.

**Meaning:**

These findings suggest that the immunogenicity rate after vaccination was lower in patients receiving dialysis and that diabetes might be a risk factor for nonresponse to vaccination.

## Teaser

This systematic review and meta-analysis assesses immunogenicity rates after COVID-19 vaccination in people with end-stage kidney disease and risk factors associated with nonresponse.

## Introduction

Adults receiving dialysis have a higher mortality risk when infected with SARS-CoV-2 than do adults not receiving dialysis.^1,2^ Because most clinical trials have not included people with end-stage kidney disease (ESKD),^3,4^ immunogenicity rates are instead assessed to determine the efficacy of SARS-CoV-2 vaccines in this population. Several studies have reported that antibody response rates vary depending on vaccination protocols, vaccine types, and populations. Old age or use of immunosuppression medication have been identified as risk factors for vaccine nonresponse.^5,6,7,8^ However, the immunogenicity rates of patients receiving dialysis have not yet been systematically reviewed, to our knowledge.

In this study, immunogenicity rates among people with ESKD after receiving SARS-CoV-2 vaccines were investigated, as were potential risk factors for vaccine nonresponse and significant differences in antibody response rates between adults receiving dialysis and those not receiving dialysis.

## Methods

### Literature Search Strategy and Eligibility Criteria

This systematic review and meta-analysis was performed in accordance with the Preferred Reporting Items for Systematic Reviews and Meta-analyses (PRISMA) reporting guideline. Regarding the systematic review, the PubMed, Embase, and Medline databases were searched for relevant articles published between January 1, 2020 and July 30, 2021 (eTable 1 in the Supplement). Keywords used to retrieve preprint articles from the *medRxiv* server included *dialysis*, *end-stage renal disease*, *SARS-CoV-2 vaccine*, *ChAdOx1 nCoV-19*, *Oxford–AstraZeneca*, *mRNA vaccines*, *BNT162b2*, and *mRNA-1273.*

We included studies on people with ESKD receiving hemodialysis or peritoneal dialysis. Other inclusion criteria were studies examining adult populations and those that reported postvaccination antibody response rates. Our search was limited to clinical trials, letters, commentaries, and preprint articles published in English after 2019. Two independent reviewers (J.-J.C. and C.-H.C.) independently assessed the titles, abstracts, and full texts (if necessary) of each publication.

### Outcome Measurement and Data Extraction

The primary outcome was postvaccination immunogenicity rates among patients with ESKD. Overall antibody response rates were analyzed if data on the neutralizing antibody were available. Otherwise, we extracted the response rates in the following sequence: anti-SARS-CoV-2 spike receptor-binding domain protein or anti-spike protein. Two reviewers (J.-J.C. and C.-H.C.) conducted the data extraction independently. We also extracted relevant variables, including the patients’ mean age, sex, dialysis vintage, diabetes diagnosis, type of vaccine received, kidney replacement modality, vaccination protocol (total doses and the interval between the first and second doses), and length of follow-up period. Studies that did and did not exclude prior SARS-CoV-2 infection were included. Any disagreements were resolved through consensus with a third author (T.H.L.).

The secondary outcome was the immunogenicity rates of patients with ESKD receiving dialysis compared with those of people without ESKD, not receiving dialysis. In most studies, the control group included health care workers in a dialysis facility.

### Risk of Bias

The quality of the cohort studies was assessed by 2 independent reviewers (J.-J.C. and C.-H.C.) using the Newcastle-Ottawa scale (NOS), which allocates a maximum of 9 points for 3 major domains: quality of the selection, comparability, and the outcome of study populations. Studies reporting scores of 7 to 9 points were considered to have low risk; 4 to 6 points, moderate risk; and less than 4 points, high risk of bias.^9^

### Statistical Analysis

The primary outcome, the proportions of postvaccination immunogenicity in the patients receiving dialysis, for the included studies was pooled using the DerSimonian-Laird random-effects model. The *I^2^* statistic was used to assess the heterogeneity of the pooled estimate, where a value greater than 0.5 was considered substantially heterogenous. To explore the potential source of heterogeneity, we conducted several subgroup analyses using mixed effects models, including (1) incomplete, complete, or booster vaccination protocols; (2) populations with prior SARS-CoV-2 infection or without prior SARS-CoV-2 infection; and (3) kidney replacement therapy modality, including hemodialysis or peritoneal dialysis. We also performed univariate random-effects meta-regression analysis using the following study-level explanatory variables: mean age, the proportion of women, mean dialysis vintage, and the prevalence of diabetes. Of note, all subgroup analysis (except regarding SARS-CoV-2 infection status) and meta-regression were performed in the selected studies that enrolled SARS-CoV-2–naive populations with complete vaccine protocols. In addition, the preprinted articles were excluded to assess the robustness of the overall result in sensitivity analysis.

The secondary outcome, immunogenicity rates of patients receiving dialysis compared with individuals not receiving dialysis, was summarized as the pooled relative risk using the DerSimonian-Laird random-effects model. The pooled estimate of secondary outcomes between the studies with complete and those with incomplete protocols was further compared using the mixed effects model. Finally, using the overall analysis of primary outcome, publication bias was assessed using visual check by funnel plot and formal statistical test by the Egger intercept test. All analyses were conducted using Comprehensive Meta-Analysis Software version 3.3.070 (Biostat). *P* values were 2-sided, and statistical significance was set at *P* < .05. Data were analyzed in August 2021.

## Results

### Literature Search

We identified 104 potentially eligible studies from the PubMed, 335 potentially eligible studies from Embase, and 46 potentially eligible studies from Medline databases. From the *medRxiv* server, 184 potentially eligible preprint studies were identified. After the titles and abstracts were screened, the full texts were reviewed to further determine study eligibility. After the exclusion of irrelevant studies, including studies not addressing the outcome of interest and studies only based on ESKD with kidney transplant population, 32 studies,^5,6,7,8,10,11,12,13,14,15,16,17,18,19,20,21,22,23,24,25,26,27,28,29,30,31^ including 6 preprint articles,^32,33,34,35,36,37^ were included in the meta-analysis (eFigure 1 in the Supplement). The extracted data are summarized in Table 1 and Table 2.

**Table 1.** Characteristics, Vaccine Type, and Vaccine Protocols of Included Studies

Study	Country	Patients, No.	KRT modality, %	Mean age	Women, %	Vaccine type	Dialysis vintage, median, y	Prior SARS-CoV-2 infection	Dose	Interval between doses
Agur et al,^17^ 2021	Israel	145	HD: 84, PD: 16	71.6	28.3	BNT162b2	3.3	No	2	3 wk
Anand et al,^32^ 2021	US	519	HD	NR	NR	JNJ-78436735, mRNA-1273, or BNT162b2	NR	Mixed	2^a^	NR
Attias et al,^6^ 2021	France	69	HD	70	21.8	BNT162b2	NR	Mixed	2	4 wk
Bertrand et al,^26^ 2021	France	9	HD	71.2	NR	BNT162b2	3.1	No	2	3 wk
Billany et al,^7^ 2021	UK	74	HD	62.1	40.4	BNT162b2 or AZD1222	NR	Mixed	1	NA
Broseta et al,^8^ 2021	Spain	100	HD	68.2	31	mRNA-1273	7	No	2	4 wk
		75	HD	74.6	34.7	BNT162b2	4	No	2	3 wk
Chan et al,^27^ 2021	US	61	HD	70	4.9	mRNA-1273	NR	Mixed	2	4 wk
Clarke et al,^33^ 2021	UK	1020	HD	NR	NR	AZD1222 or BNT162b2	NR	Mixed	2	9 wk (median 63 d)^b^
Danthu et al,^10^ 2021	France	75	HD	73.5	42.7	BNT162b2	5.1	No	2	4 wk
Duarte et al,^34^ 2021	Portugal	42	HD	75.1	40.5	BNT162b2	NR	No	2	3 wk
		25	PD	60.5	28	BNT162b2	NR	No	2	3 wk
Ducloux et al,^14^ 2021	France	45	HD	NR	NR	BNT162b2	NR	No	2^c^	NR
Frantzen et al,^28^ 2021	France	244	HD	76	30.3	BNT162b2	NR	Mixed	2	3 wk
Frantzen et al,^15^ 2021 (3rd boost dose cohort)	France	88	HD	76	27.3	BNT162b2	NR	No	3	2-3 mo after dose 2
Goupil et al,^11^ 2021	Canada	131	HD	70 (naive)	33.6	BNT162b2	3.8	No	1	NA
		19	HD	76 (infected)	47.3		3.4	Yes	1	NA
Grupper et al,^20^ 2021	Israel	56	HD	74	25	BNT162b2	3.5	Mixed	2	3 wk
Lacson et al,^29^ 2021	US	186	HD	67.9	47.3	BNT162b2 or mRNA-1273	4.8	Mixed	2	NR
Jahn et al,^22^ 2021	Germany	72	HD	68	56.9	BNT162b2	4.3	No	2	4 wk
Lensy et al,^12^ 2021	Multiple	27	HD: 85, PD: 15	63.4	44.4	BNT162b2 or AZD1222	2.2	No	1	NA
Longlune et al,^16^ 2021	France	98	HD: 80 PD: 20	NR	NR	BNT162b2	NR	No	2^c^	4 wk
Rincon-Arevalo et al,^21^ 2021	Germany	44	HD: 91, PD: 9	NR^d^	36.4	BNT162b2	5.5	Mixed	2	3 wk
Rodríguez-Espinosa et al,^19^ 2021	Spain	32	PD	63.9	65.6	mRNA-1273	NR	No	2	4 wk
Sattler et al,^25^ 2021	Germany	26	HD	67.4	34.6	BNT162b2	6.9	No	2	3 wk
Schrezenmeier et al,^18^ 2021	Germany	36	HD: 94.4, PD: 5.6	74	30.5	BNT162b2	5	No	2	3 wk
Simon et al,^30^ 2021	Austria	81	HD	67	28.4	BNT162b2	NR	No	2	3 wk
Speer et al,^23^ 2021	Germany	22	HD	74	45.5	BNT162b2	5	No	2	3 wk (19-22 d)
Strengert et al,^35^ 2021	Germany	81	HD	69	42	BNT162b2	3.8	No	2	3 wk
Torreggiani et al,^13^ 2021	France	95	HD	69.5	42.1	BNT162b2	2.4	Mixed	1	NA
Weigert et al,^36^ 2021	Portugal	143	HD	72	41.3	BNT162b2	3.8	No	2	3 wk
Yanay et al,^24^ 2021	Israel	160	HD	69	36.9	BNT162b2	3.2	No	2	NR
Yau et al,^37^ 2021 (2 dose cohort)	Canada	72	HD	75	41.7	BNT162b2	NR	Mixed	2	3 wk
Yau et al,^37^ 2021 (1 dose cohort)	Canada	66	HD	72	27.2	BNT162b2	NR	Mixed	1	NA
Yi et al,^5^ 2021	US	26	NR	NR	NR	BNT162b2 or mRNA-1273	NR	No	2	NR
Zitt et al,^31^ 2021	Austria	47	HD	67.6	34.0	BNT162b2	2.7	No	2	4 wk

Abbreviations: HD, hemodialysis; KRT, kidney replacement therapy; PD, peritoneal dialysis; NA, not applicable; NR, not reported; UK, United Kingdom.

^a^ Patients who received JNJ-78436735 received 1 dose.

^b^ Vaccine interval was NR in patients who had prior SARS-CoV-2 infection.

^c^ Proportion of participants received third boost dose vaccine.

^d^ HD population median age: 69 years, PD population median age: 70.5 years.

**Table 2.** Outcomes of the Included Studies

Study	Antibodies outcome	Follow up period	Dose	Patients receiving dialysis		Participants not receiving dialysis	
				No.	Responders, No. (%)	No.	Responders, No. (%)
Agur et al,^17^ 2021	Anti-S IgG	2-6 wk	2	HD: 122 ; PD: 23	HD: 114 (93.4); PD: 22 (95.7)	0	NA
Anand et al,^32^ 2021	Anti-RBD IgG	2-4 wk^a^	2	610	474 (77.7)	0	NA
Attias et al,^6^ 2021	Anti-S IgG	3 wk	2	64	55 (85.9)	0	NA
		4 wk	1	69	23 (33.3)		
Bertrand et al,^26^ 2021	Anti-S IgG	4 wk	2	9	8 (88.9)	0	NA
		3 wk	1	9	1 (11.1)		
Billany et al,^7^ 2021	Anti-RBD IgG	4 wk (mean 27.8 d)	1	95	75 (79.8)	0	NA
Broseta et al,^8^ 2021 (mRNA-1273)	Anti-RBD IgG	3 wk	2	100	98 (98)	0	NA
Broseta et al,^8^ 2021 (BNT162b2)	Anti-RBD IgG	3 wk	2	75	69 (92)	0	NA
Chan et al,^27^ 2021	Anti-RBD IgG	1 wk	2	61	58 (95.1)	0	NA
Clarke et al,^33^ 2021	Anti-S IgG	5-6 wk (median 39-41 d)^b^	2	1020	938 (92)	0	NA
Danthu et al,^10^ 2021	Anti-S IgG	36 d	2	79	59 (78.7)	7	7 (100)
Duarte et al,^34^ 2021	Anti-S IgG	3 wk	2	HD: 42; PD: 25	HD: 36 (85.7); PD: 25 (100)	0	NA
			1	HD: 42; PD: 25	HD: 21 (50); PD: 22 (88)	0	NA
Ducloux et al,^14^ 2021	Anti-RBD IgG	NR	2	45	40 (88.9)	0	NA
Ducloux et al,^14^ 2021	Anti-RBD IgG	NR	3	45	42 (93.3)	0	NA
Frantzen et al,^28^ 2021	Anti-S IgG	4 wk	2	244	221 (90.6)	0	NA
Frantzen et al,^15^ 2021 (3rd boost dose cohort)	Anti-S IgG	4 wk	3	88	86 (97.7)	0	NA
Goupil et al,^11^ 2021	Anti-RBD IgG	4 wk	1	SARS-CoV-2 naive: 131; previous infection: 19	SARS-CoV-2 naive: 75 (57.3); previous infection: 16 (84.2)	0	NA
Grupper et al,^20^ 2021	Anti-RBD IgG	30 d (median)	2	56	54 (96.5)	95	95 (100)
Lacson et al,^29^ 2021	Anti-RBD IgG	2 wk	2	186	165 (88.7)	0	NA
Jahn et al,^22^ 2021	Anti-S IgG	2 wk	2	72	67 (93.1)	16	16 (100)
Lensy et al,^12^ 2021	Anti-S IgG	2 wk	1	27	8 (29.6)	14	8 (57.1)
Longlune et al,^16^ 2021	Anti-S IgG	4 wk	2	HD: 78; PD: 20	HR: 64 (82.1); PD: 17 (85)	0	NA
		4 wk	1	HD: 80; PD: 24	HD: 17 (21.3); PD: 10 (41.7)	0	NA
		1 mo	3	HD: 77	69 (89.6)	0	NA
Rincon-Arevalo et al,^21^ 2021	Anti-S IgG	1 wk	2	44	31 (70.5)	25	25 (100)
Rodríguez-Espinosa et al,^19^ 2021	Anti-RBD IgG	3 wk	2	32	31 (96.9)	0	NA
		4 wk	1	32	20 (62.5)	0	NA
Sattler et al,^25^ 2021	Anti-S IgG	8 d	2	26	22 (84.6)	39	39 (100)
Schrezenmeier et al,^18^ 2021	Anti-S IgG	3-4 wk	2	36	32 (88.9)	44	NR
Simon et al,^30^ 2021	Anti-RBD	3 wk	2	81	59 (72.8)	80	80 (100)
Speer et al,^23^ 2021	Neutralizing antibodies	20 d (median)	2	22	14 (62.6)	46	46 (100)
		18 d (median)	1	22	4 (18.2)	46	43 (93.5)
Strengert et al,^35^ 2021	Anti-S IgG	3 wk	2	81	77 (95.1)	34	NR
Torreggiani et al,^13^ 2021	Anti-S IgG	3 wk	1	95	35 (36.8)	0	NA
Weigert et al,^36^ 2021	Anti-S IgG	3 wk	2	143	130 (91.0)	143	136 (95.1)
		NA	1	143	42 (29.4)	143	71 (49.7)
Yanay et al,^24^ 2021	Anti-S IgG	3-5 wk	2	160	144 (90)	132	132 (100)
Yau et al,^37^ 2021 (2 dose cohort)	Anti-RBD IgG	2 wk	2	72	63 (87.5)	35	35 (100)
		3 wk	1	76	31 (40.8)	0	NA
Yau et al,^37^ 2021 (1 dose cohort)	Anti-RBD IgG	4 wk	1	66	33 (50)	0	NA
Yi et al,^5^ 2021	Anti-S IgG	NR	2	31	26 (83.9)	0	NA
Zitt et al,^31^ 2021	Anti-S IgG	4 wk	2	47	46 (97.9)	0	NA
		4 wk	1	50	21 (42)	0	NA

Abbreviations: HD, hemodialysis; IgG, immunoglobin G; PD, peritoneal dialysis; NA, not applicable; NR, not reported; RBD, receptor-binding domain.

^a^ 2 Weeks (mRNA), >2 weeks (JNJ-78436735), Median, 29 days.

^b^ Vaccine interval was NR in prior SARS-CoV-2 infection population.

### Study Characteristics

A total of 4917 participants were included in this meta-analytic study, and 4 vaccines (JNJ-78436735 [Janssen], mRNA-1273 [Moderna], BNT162b2 [Pfizer-BioNTech], and AZD1222 [AstraZeneca]) were administered. Most participants received the BNT162b2 vaccine. All enrolled participants did not receive a mixture of different SARS-CoV-2 vaccines. Six preprint articles were selected from the *medRxiv* server and another 5 studies from enrolled studies only reported the immunogenicity rates of patients receiving dialysis who did not complete the vaccination protocol (ie, those who received only 1 dose of the BNT162b, AZD1222, or mRNA-1273 vaccines).^7,11,12,13,37^ Other articles reported an antibody response rate after the second dose with or without reporting the response rate after the first dose. Three studies discussed immunogenicity rates after the booster shot.^14,15,16^ The mean age of the participants ranged from 60.5 to 76 years (Table 1). Hemodialysis was the predominant kidney replacement therapy modality. Five studies enrolled people receiving mixed peritoneal dialysis and hemodialysis,^12,16,17,18,34^ and only 1 study^19^ enrolled patients only receiving peritoneal dialysis (Table 1). Few studies reported the dialysis adequacy in the enrolled population, and all of them were noted with a Kt/V exceeding 1.3. The mean dialysis vintage varied from 1.7 to 7 years, and all included studies had a low to moderate risk of bias (NOS score: 5-8) (eTable 2 in the Supplement).

### Primary and Secondary Outcomes

**Figure 1.**
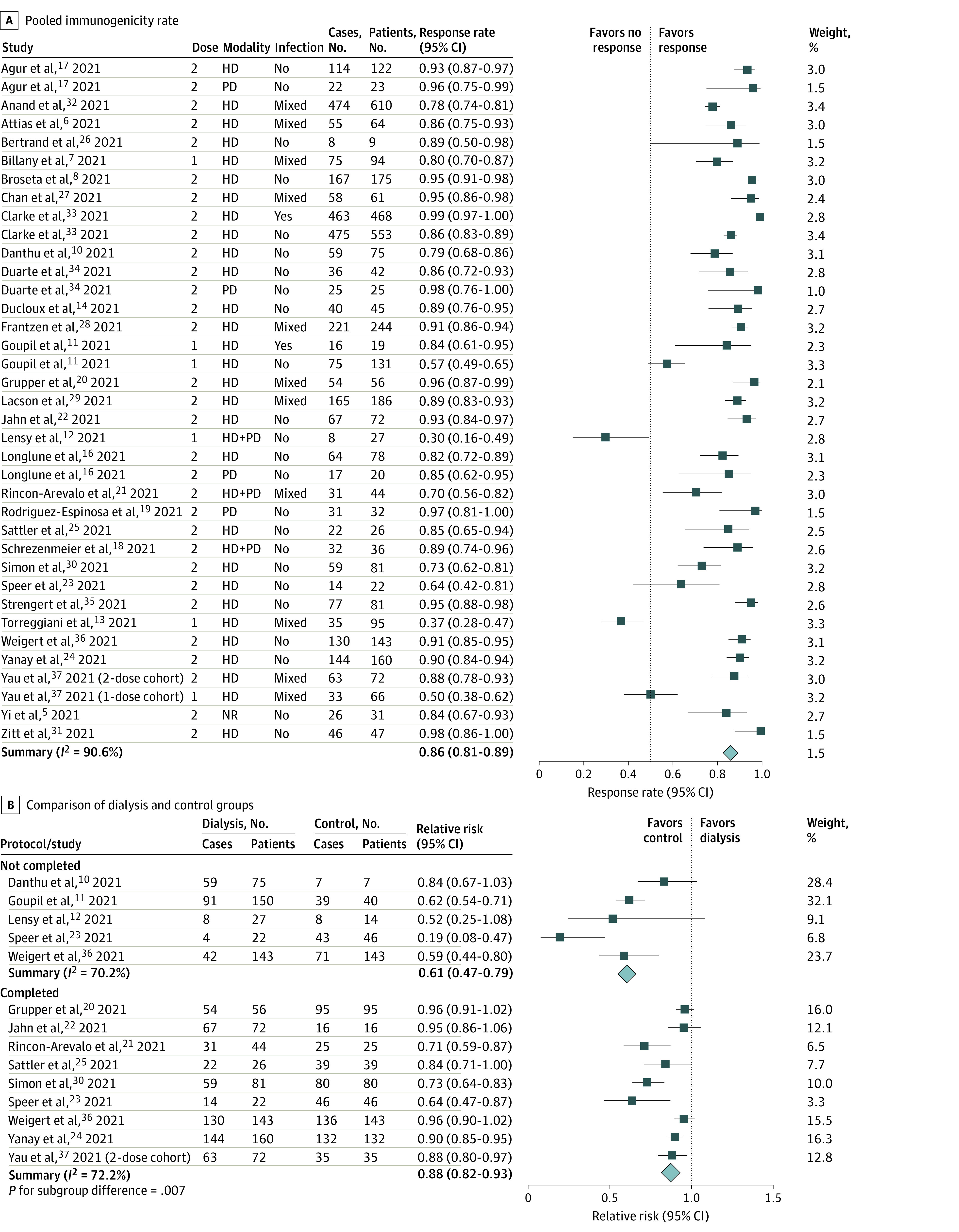
Forest Plots of Immunogenicity Rates

The meta-analysis revealed that the overall immunogenicity rate in patients receiving dialysis was 86% (95% CI, 81%-89%) with high heterogeneity (*I*^2^ = 90.6%) (Figure 1A). We also performed meta-analysis to determine whether the antibody response rate was significantly different between patients receiving dialysis and individuals not receiving dialysis. Immunogenicity rates after the first dose (relative risk [RR], 0.61; 95% CI, 0.47-0.79; *I*^2^ = 70.2%) and second dose (RR, 0.88; 95% CI, 0.82-0.93; *I*^2^ = 72.2%) were significantly lower in the dialysis group than in the control group. Noticeably, the lower response rate of the patients receiving dialysis, relative to those not receiving dialysis, was less apparent in the second dose than that in the first dose (*P* = .007) (Figure 1B).

### Sensitivity Analysis

We conducted a sensitivity analysis that excluded the preprinted and unpublished articles. The result was highly consistent to that of the primary analysis, with the pooled immunogenicity rate of 85% (95% CI, 79%-90%) along with high heterogeneity (*I*^2^ = 89.8%) (eFigure 2 in the Supplement).

### Subgroup Analysis and Meta-regression

**Figure 2.**
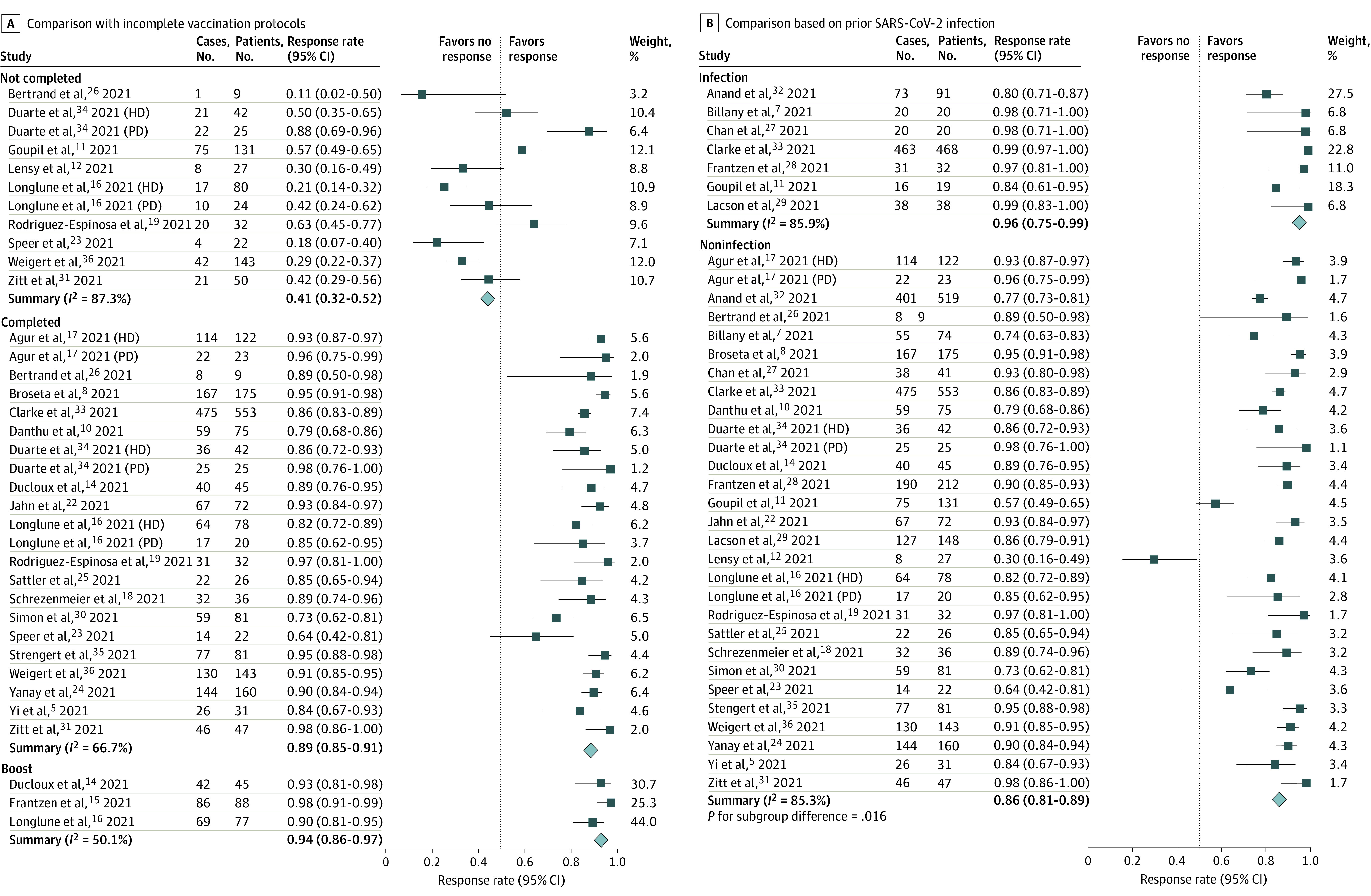
Forest Plots of Immunogenicity Rates By Vaccine Protocol Status

Subgroup analysis was performed to identify the potential source of heterogeneity. The response rate was the lowest in patients without complete vaccination protocols (41%; 95% CI, 32%-52%; *I*^2^ = 87.3%), followed by in those with complete vaccination protocols (89%; 95% CI, 85%-91%; *I*^2^ = 66.7%) and was highest in those with third booster vaccine protocols (94%; 95% CI, 86%-97%; *I*^2^ = 50.1%; *P* < .001) Figure 2A). The postvaccination immune response rates of participants with a history of SARS-CoV-2 infection was significantly higher than those without such a history (96%; 95% CI, 95%-99%; vs 86%; 95% CI, 81%-89%; *P* = .02; Figure 2B). No significant difference in the response rate was detected between hemodialysis and peritoneal dialysis (87%; 95% CI, 83%-90%; vs 94%; 95% CI, 84%-98%; *P* = .15) (eFigure 3 in the Supplement).

**Figure 3.**
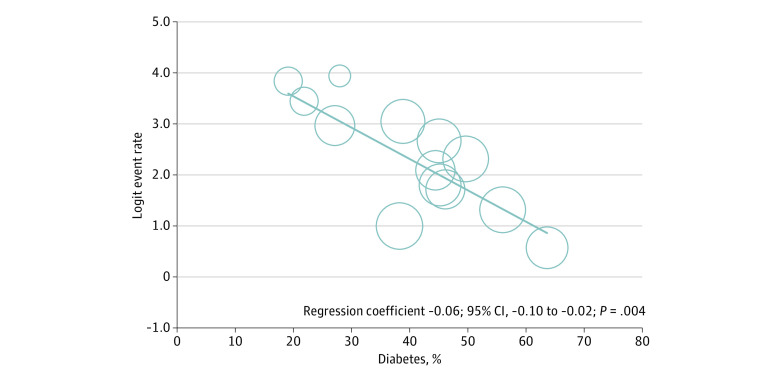
Association of Diabetes Prevalence and Immunogenicity Rates in the SARS-CoV-2–Naive Dialysis Population With Complete Vaccine Protocol

The meta-regression analysis showed that a higher prevalence of diabetes was significantly correlated with a lower immune response rate (regression coefficient, −0.06; 95% CI, −0.10 to −0.02; *P* = .004) (Figure 3). No significant associations between mean age, proportion of women, dialysis vintage, and response rate were observed (eFigures 4-6 in the Supplement).

### Publication Bias

The funnel plot revealed some asymmetry, which implied that there was a problem of publication bias (eFigure 7 in the Supplement). The Egger test also revealed a significant result (*P* = .04) which indicated the existence of publication bias in this meta-analysis.

## Discussion

This systematic review and meta-analysis found 4 notable results. First, the overall immunogenicity rate in the analyzed studies was 86%. Second, the immunogenicity rates after the first dose and the second dose were both significantly lower in individuals receiving dialysis than in those not receiving dialysis, and this difference was smaller after 2 doses. Third, the immunogenicity rates were significantly higher in those with a prior history of SARS-CoV-2 infection, and the immunogenicity rates increased significantly after the second and third booster dose compared with the immunogenicity rates observed after the first dose. Furthermore, a significant inverse association between diabetes prevalence and response rate was noted.

Several studies have reported that people receiving dialysis have lower immune response rates after SARS-CoV-2 vaccination than do those not receiving dialysis.^10,11,12,20,21,22,23,24,25,30,36^ This result is inconsistent with the previous studies that have demonstrated that patients with ESKD who are receiving dialysis have a lower immune response to the hepatitis B virus vaccine compared with the general population.^38,39^ Lower mean antibody titer and poor T-cell immune responses relative to individuals not receiving dialysis were also observed in those receiving dialysis.^23^ A study by Speer et al^23^ has indicated that patients receiving dialysis had not only a lower probability of producing antispike antibodies but also a lower probability of producing overthreshold levels of neutralizing antibodies. In this study, we found that immunogenicity rates after the first and the second dose were lower among individuals receiving dialysis than in those not receiving dialysis. Moreover, this difference in response rates became significantly smaller after the second dose. Our findings suggest that in the dialysis population, scheduled, earlier second vaccination, not a delayed second COVID-19 vaccine dose protocol, may be better.

Risk factors for nonresponse among patients receiving dialysis included old age,^6,7,8^ nonresponse to hepatitis B vaccination,^10^ low serum albumin levels,^10,17^ dialysis inadequacy,^10^ and use of immunosuppressants.^16,26^ The use of old age as a risk factor for vaccine nonresponse was inconsistent in the reviewed studies, and some trials did not determine that old age was a significant risk factor for poor response.^16,23^ Speer et al^23^ noted an inverse linear association between age and immune response rate in a healthy control group and no age-related differences in neutralizing antibody production.^20^ In this study, we detected no inverse linear association between age and immunogenicity rates.

Diabetes was considered a risk factor for immune system dysfunction and nonresponse after vaccination according to previous studies.^40,41^ The combination of diabetes and ESKD was demonstrated as a risk factor for hyporesponsiveness after hepatitis B vaccination.^42,43^ In this study, we found that prevalence of diabetes was inversely associated with immune response rate after SARS-CoV-2 vaccination. Further larger, well-designed studies are needed to examine this association.

### Limitations

Our study has several limitations. First, in the examined studies, vaccination efficacy was based on the immunobridging approach, which relies on humoral immunity. However, after vaccination, increased intracellular production of S protein also primes both CD8^+^ and CD4^+^ T cells to differentiate into effector and memory subsets of inflammatory and cytotoxic mediators and CD4^+^ T helper cells, which promote B cell differentiation into antibody-secreting plasma cells. The ability of antibodies to neutralize the mutated virus might decrease, but cellular immunity remains activated during infection by the mutated virus.^44^ However, because the assessment of cellular immunity was not well established, the comparison conducted in this meta-analysis cannot completely reflect the protective effect of vaccination. Furthermore, some studies examining mixed SARS-CoV-2 vaccines did report the immunogenicity by measuring the antibody levels or T cell reactivity by interferon γ release assay.^45,46^ Real-world evidence of mixed vaccines protocols is lacking. Ethical concerns are challenging the use of placebos in phase 3 randomized clinical trials on vaccines. Therefore, immunobridging research provides a comparable platform for various vaccines. Second, we only evaluated the immune response rate after the administration of the third booster dose. A study by Frantzen et al^15^ found increases in the antibody levels of patients who had received the booster shot. Further studies are required to examine the immune response and clinical protective effect of the booster dose in patients receiving dialysis.

## Conclusions

This systematic review and meta-analysis found that patients with ESKD had a pooled postvaccine immune response rate of 86%. Compared with the nondialysis group, patients receiving dialysis had a lower probability of producing an antibody response after receiving the first dose and second dose of a COVID-19 vaccine. Furthermore, this difference between nondialysis and dialysis populations became statistically smaller after the second dose. Scheduling the second vaccine dose without delay might be preferable in patients receiving dialysis. Prevalence of diabetes had an inverse linear association with the immune response rate. Further investigations of immune response and side effects of SARS-CoV-2 vaccines in patients receiving dialysis, as well as the benefits and real world clinical efficacy of different vaccine protocols, different types of vaccine, are warranted.

## Notes

Supplement. **eTable 1.** Search Strategy and Result **eTable 2.** Newcastle-Ottawa Scale Assessment of the Included Studies **eFigure 1.** Study Inclusion Flowchart **eFigure 2.** Sensitivity Analysis of Pooled Immunogenicity Rate in Patients Receiving Dialysis After Excluding the Preprint Articles **eFigure 3.** Subgroup Analysis Comparing the Pooled Immunogenicity Rates in Patients Receiving Hemodialysis or Peritoneal Dialysis in SARS-CoV-2–Naive Dialysis Population With Complete Vaccine Protocol **eFigure 4.** Associations Between Age and Immunogenicity Rates in SARS-CoV-2–Naive Dialysis Population with Complete Vaccine Protocol **eFigure 5.** Associations Between Proportion of Women and Immunogenicity Rates in SARS-CoV-2–Naive Dialysis Population With Complete Vaccine Protocol **eFigure 6.** Associations Between Dialysis Vintage and Immunogenicity Rates in SARS-CoV-2–Naive Dialysis Population With Complete Vaccine Protocol **eFigure 7.** Funnel Plot Illustrating Publication Bias
