# Supplementary material for: Immunogenicity Rates After SARS-CoV-2 Vaccination in People With End-stage Kidney Disease: A Systematic Review and Meta-analysis
Source: JAMA Netw Open. 2021 Oct 28;4(10):e2131749. doi: 10.1001/jamanetworkopen.2021.31749 (PMC8554642; doi:10.1001/jamanetworkopen.2021.31749)
Supplement: Supplement. — eTable 1. Search Strategy and Result eTable 2. Newcastle-Ottawa Scale Assessment of the Included Studies eFigure 1. Study Inclusion Flowchart eFigure 2. Sensitivity Analysis of Pooled Immunogenicity Rate in Patients Receiving Dialysis After Excluding the Preprint Articles eFigure 3. Subgroup Analysis Comparing the Pooled Immunogenicity Rates in Patients Receiving Hemodialysis or Peritoneal Dialysis in SARS-CoV-2–Naive Dialysis Population With Complete Vaccine Protocol eFigure 4. Associations Between Age and Immunogenicity Rates in SARS-CoV-2–Naive Dialysis Population with Complete Vaccine Protocol eFigure 5. Associations Between Proportion of Women and Immunogenicity Rates in SARS-CoV-2–Naive Dialysis Population With Complete Vaccine Protocol eFigure 6. Associations Between Dialysis Vintage and Immunogenicity Rates in SARS-CoV-2–Naive Dialysis Population With Complete Vaccine Protocol eFigure 7. Funnel Plot Illustrating Publication Bias [file jamanetwopen-e2131749-s001.pdf]

Supplemental Online Content

Chen JJ, Lee TH, Tian YC, Lee CC, Fan PC, Chang CH. Immunogenicity rates after SARS-CoV-2 vaccination in people with end-stage kidney disease: a systematic review and meta-analysis. *JAMA Netw Open*. 2021;4(10):e2131749. doi:10.1001/jamanetworkopen.2021.31749

eTable 1. Search Strategy and Result
eTable 2. Newcastle-Ottawa Scale Assessment of the Included Studies
eFigure 1. Study Inclusion Flowchart
eFigure 2. Sensitivity Analysis of Pooled Immunogenicity Rate in Patients Receiving Dialysis After Excluding the Preprint Articles
eFigure 3. Subgroup Analysis Comparing the Pooled Immunogenicity Rates in Patients Receiving Hemodialysis or Peritoneal Dialysis in SARS-CoV-2–Naive Dialysis Population With Complete Vaccine Protocol
eFigure 4. Associations Between Age and Immunogenicity Rates in SARS-CoV-2–Naive Dialysis Population with Complete Vaccine Protocol
eFigure 5. Associations Between Proportion of Women and Immunogenicity Rates in SARS-CoV-2–Naive Dialysis Population With Complete Vaccine Protocol
eFigure 6. Associations Between Dialysis Vintage and Immunogenicity Rates in SARS-CoV-2–Naive Dialysis Population With Complete Vaccine Protocol
eFigure 7. Funnel Plot Illustrating Publication Bias

This supplemental material has been provided by the authors to give readers additional information about their work.

© 2021 Chen JJ et al. *JAMA Network Open.*

eTable 1. Search Strategy and Result

**Pubmed through July 30st, 2021**	
#1	((((((((((Kidney Failure, Chronic[MeSH Terms]) OR (Renal Dialysis[MeSH Terms])) OR (Renal Insufficiency, Chronic[MeSH Terms])) OR (End stage renal disease)) OR (End stage kidney disease)) OR (Chronic renal failure)) OR (Chronic kidney disease)) OR (Chronic renal insufficiency)) OR (Chronic kidney failure)) OR (dialysis)) OR (hemodialysis)) OR (peritoneal dialysis)
#2	((COVID-19) OR (SARS-CoV-2)) AND (coronavirus)
#3	((((COVID-19 Vaccines[MeSH Terms]) OR (Immunogenicity, Vaccine[MeSH Terms])) OR (Spike Glycoprotein, Coronavirus[MeSH Terms])) OR (vaccine)) OR (vaccination)
#4	1# AND 2# AND #3 + Filters: from 2019 - 2021 , Result: 104
**Medline through July 30st, 2021**	
#1	Renal Dialysis/ or Dialysis/
#2	end stage renal disease.mp. or Kidney Failure, Chronic/
#3	AstraZeneca.mp.
#4	mRNA vaccine.mp.
#5	Tozinameran.mp.
#6	Comirnaty.mp.
#7	Moderna.mp.
#8	Janssen.mp.
#9	bnt162b2.mp.
#10	pfizer-biontech.mp.
#11	mrna-1273.mp.
#12	COVID-19 vaccine.mp. or COVID-19 Vaccines/
#13	#1 AND #2

© 2021 Chen JJ et al. *JAMA Network Open.*

#14	#3 or #4 or #5 or #6 or #7 or #8 or #9 or #10 or #11 or #12
#15	#13 AND #14, Result: 46
**EMbase through July 30st, 2021**	
#1	'dialysis'/exp OR dialysis'
#2	'hemodialysis'/exp OR hemodialysis'
#3	'peritoneal dialysis'/exp OR 'peritoneal dialysis' OR (peritoneal AND ('dialysis'/exp OR dialysis))
#4	'chronic kidney failure'/exp OR 'chronic kidney failure' OR (chronic AND ('kidney'/exp OR kidney) AND ('failure'/exp OR failure))
#5	#1 OR #2 OR #3 OR #4
#6	'sars-cov-2 vaccine'/exp OR 'sars-cov-2 vaccine'
#7	'mrna-1273 vacccine' /exp OR 'mrna-1273 vacccine'
#8	'chadox1 ncov 19' /exp OR 'chadox1 ncov 19'
#9	'bnt162b2' /exp OR 'bnt162b2'
#10	'astrazeneca'/exp OR 'astrazeneca'
#11	('mrna'/exp OR mrna) AND ('vaccine'/exp OR vac cine)
#12	'tozinameran'/exp OR tozinameran
#13	'comirnaty'/exp OR comirnaty
#14	'moderna'/exp OR moderna
#15	'pfizer biontech'
#16	'ad26.cov2.s'/exp OR ad26.cov2.s
#17	#6 OR #7 OR #8 0R #9 OR #10 OR #11 OR #12 OR #13 OR #14 OR #15 OR #16
#18	#5 AND #17, Result 768 articles
#19	#18 AND (2020:py OR 2021:py) , Result 335 articles

© 2021 Chen JJ et al. *JAMA Network Open.*

eTable 2. Newcastle-Ottawa Scale Assessment of the Included Studies

study	Representativeness of the exposed cohort (1)	Selection of the non exposed cohort (1)	Ascertainment of exposure (1)	Demonstration that outcome of interest was not present at start of study (1)	Comparability of cohorts on the basis of the design or analysis (2)	Assessment of outcome (1)	Was follow-up long enough for outcomes to occur (1)	Adequacy of follow up of cohorts (1)	NOS_score
Agur,2021	1	0	1	1	0	1	1	1	6
Anand,2021	1	0	1	0	0	1	1	1	5
Attias, 2021	1	0	1	0	0	1	1	1	5
Bertrand,2021	1	0	1	1	0	1	1	1	6
Billany,2021	1	0	1	0	0	1	1	1	5
Broseta,2021	1	0	1	1	0	1	1	1	6
Chan, 2021	1	0	1	0	0	1	1	1	5
Clarke, 2021	1	5	1	0	0	1	1	1	5
Danthu,2021	1	1	1	1	1	1	1	1	8
Duarte, 2021	1	0	1	1	0	1	1	1	6
Ducloux,2021	1	0	1	0	0	1	1	1	5
Frantzen,2021	1	0	1	0	0	1	1	1	5
Frantzen,2021 (3rd boost dose cohort)	1	0	1	1	0	1	1	1	6
Goupil,2021	1	0	1	1	0	1	1	1	6
Grupper,2021	1	1	1	0	1	1	1	1	7
Lacson,2021	1	0	1	0	0	1	1	1	5
Jahn, 2021	1	1	1	1	1	1	1	1	8

© 2021 Chen JJ et al. *JAMA Network Open.*

Lensy,2021	1	1	1	1	1	1	1	1	8
Longlune,2021	1	0	1	1	0	1	1	1	6
Rincon-Arevalo,2021	1	1	1	0	1	1	1	1	7
Rodríguez-Espinosa,2021	1	0	1	1	0	1	1	1	6
Sattler, 2021	1	1	1	1	1	1	1	1	8
Schrezenmeier, 2021	1	1	1	1	1	1	1	1	8
Simon, 2021	1	1	1	1	1	1	1	1	8
Speer,2021	1	1	1	1	1	1	1	1	8
Strengert,2021	1	1	1	1	1	1	1	1	8
Torreggiani,2021	1	0	1	0	0	1	1	1	5
Weigert, 2021	1	1	1	1	1	1	1	1	8
Yanay, 2021	1	1	1	1	1	1	1	1	8
Yau,2021	1	1	1	0	1	1	1	1	7
Yi, 2021	1	0	1	1	0	1	1	1	6
Zitt, 2021	1	0	1	1	0	1	1	1	6

© 2021 Chen JJ et al. *JAMA Network Open.*

eFigure 1. Study Inclusion Flowchart

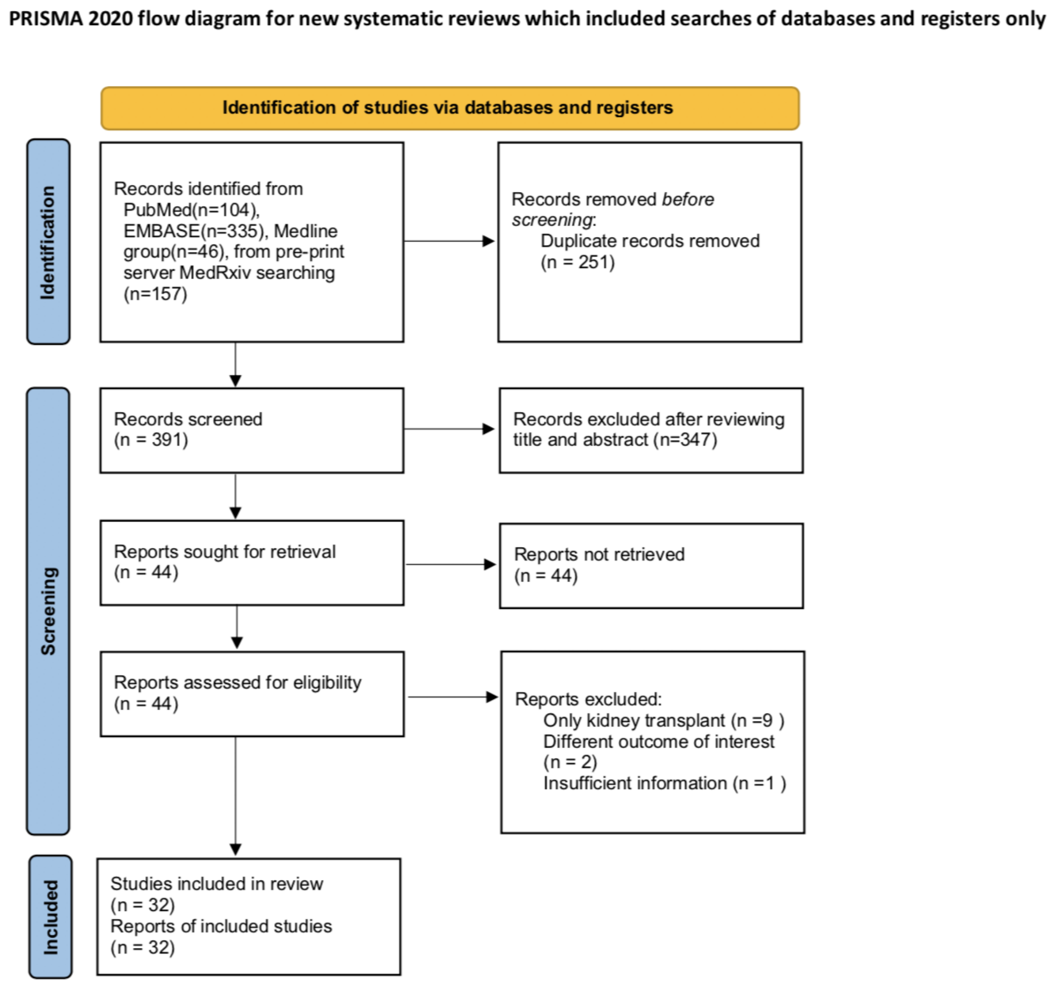

From: Page MJ, McKenzie JE, Bossuyt PM, Boutron I, Hoffmann TC, Mulrow CD, et al. The PRISMA 2020 statement: an updated guideline for reporting systematic reviews. BMJ 2021;372:n71. doi: 10.1136/bmj.n71

For more information, visit: http://www.prisma-statement.org/

eFigure 2. Sensitivity Analysis of Pooled Immunogenicity Rate in Patients Receiving Dialysis After Excluding the Preprint Articles

Study	Dose	Modality	Infection	n/N	Response rate (95% CI)	Weight (%)
Agur	2	HD	No	114 / 122	0.93 (0.87 to 0.97)	3.9
Agur	2	PD	No	22 / 23	0.96 (0.75 to 0.99)	2.2
Attias	2	HD	Mixed	55 / 64	0.86 (0.75 to 0.93)	3.9
Bertrand	2	HD	No	8 / 9	0.89 (0.50 to 0.98)	2.1
Billany	1	HD	Mixed	75 / 94	0.80 (0.70 to 0.87)	4.1
Broseta	2	HD	No	167 / 175	0.95 (0.91 to 0.98)	3.9
Chan	2	HD	Mixed	58 / 61	0.95 (0.86 to 0.98)	3.3
Danthu	2	HD	No	59 / 75	0.79 (0.68 to 0.86)	4.1
Ducloux	2	HD	No	40 / 45	0.89 (0.76 to 0.95)	3.6
Frantzen	2	HD	Mixed	221 / 244	0.91 (0.86 to 0.94)	4.2
Goupil	1	HD	Yes	16 / 19	0.84 (0.61 to 0.95)	3.2
Goupil	1	HD	No	75 / 131	0.57 (0.49 to 0.65)	4.3
Grupper	2	HD	Mixed	54 / 56	0.96 (0.87 to 0.99)	3.0
Lacson	2	HD	Mixed	165 / 186	0.89 (0.83 to 0.93)	4.2
Jahn	2	HD	No	67 / 72	0.93 (0.84 to 0.97)	3.7
Lensy	1	HD+PD	No	8 / 27	0.30 (0.16 to 0.49)	3.8
Longlune	2	HD	No	64 / 78	0.82 (0.72 to 0.89)	4.1
Longlune	2	PD	No	17 / 20	0.85 (0.62 to 0.95)	3.2
Rincon-Arevalo	2	HD+PD	Mixed	31 / 44	0.70 (0.56 to 0.82)	4.0
Rodríguez-Espinosa	2	PD	No	31 / 32	0.97 (0.81 to 1.00)	2.2
Sattler	2	HD	No	22 / 26	0.85 (0.65 to 0.94)	3.4
Schrezenmeier	2	HD+PD	No	32 / 36	0.89 (0.74 to 0.96)	3.5
Simon	2	HD	No	59 / 81	0.73 (0.62 to 0.81)	4.2
Speer	2	HD	No	14 / 22	0.64 (0.42 to 0.81)	3.7
Torreggiani	1	HD	Mixed	35 / 95	0.37 (0.28 to 0.47)	4.2
Yanay	2	HD	No	144 / 160	0.90 (0.84 to 0.94)	4.1
Yi	2	NR	No	26 / 31	0.84 (0.67 to 0.93)	3.6
Zitt	2	HD	No	46 / 47	0.98 (0.86 to 1.00)	2.2
**Summary (*I*-square = 89.8%)**					**0.85 (0.79 to 0.90)**	

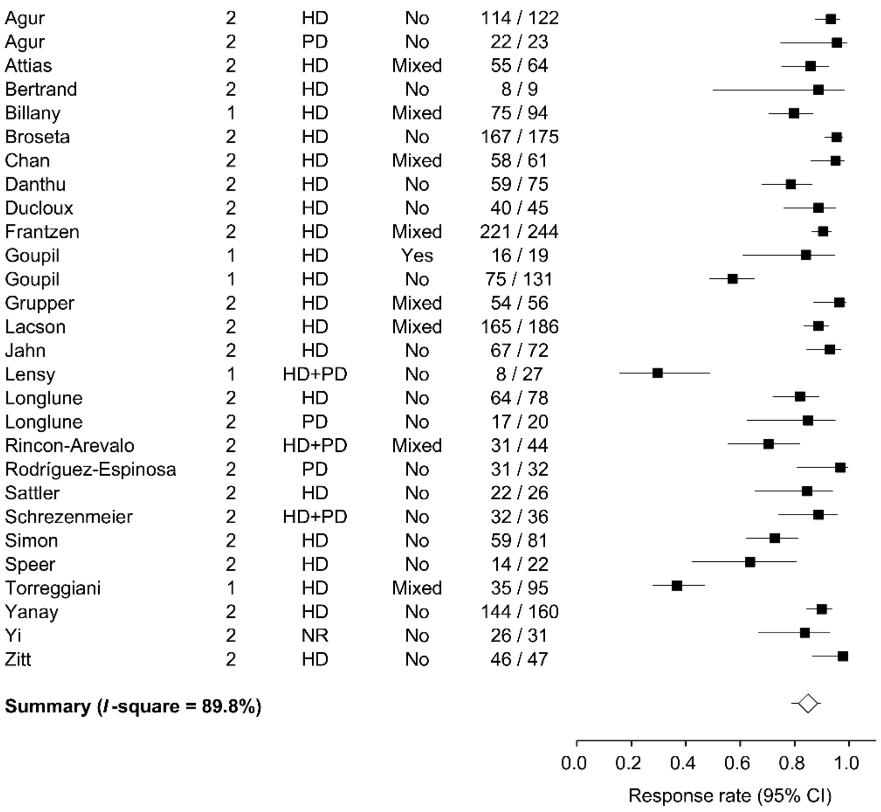

© 2021 Chen JJ et al. *JAMA Network Open.*

eFigure 3. Subgroup Analysis Comparing the Pooled Immunogenicity Rates in Patients Receiving Hemodialysis or Peritoneal Dialysis in SARS-CoV-2–Naive Dialysis Population With Complete Vaccine Protocol

Modality / Study	n/N	Response rate (95% CI)	Weight (%)
**HD**			
Agur	114 / 122	0.93 (0.87 to 0.97)	4.9
Anand	401 / 519	0.77 (0.73 to 0.81)	7.1
Bertrand	8 / 9	0.89 (0.50 to 0.98)	1.4
Billany	55 / 74	0.74 (0.63 to 0.83)	5.8
Broseta	167 / 175	0.95 (0.91 to 0.98)	4.9
Chan	38 / 41	0.93 (0.80 to 0.98)	3.1
Clarke	475 / 553	0.86 (0.83 to 0.89)	7.0
Danthu	59 / 75	0.79 (0.68 to 0.86)	5.6
Duarte	36 / 42	0.86 (0.72 to 0.93)	4.2
Ducloux	40 / 45	0.89 (0.76 to 0.95)	4.0
Frantzen	190 / 212	0.90 (0.85 to 0.93)	6.2
Jahn	67 / 72	0.93 (0.84 to 0.97)	4.1
Lacson	127 / 148	0.86 (0.79 to 0.91)	6.1
Longlune	64 / 78	0.82 (0.72 to 0.89)	5.5
Sattler	22 / 26	0.85 (0.65 to 0.94)	3.5
Simon	59 / 81	0.73 (0.62 to 0.81)	5.9
Speer	14 / 22	0.64 (0.42 to 0.81)	4.2
Strengert	77 / 81	0.95 (0.88 to 0.98)	3.7
Weigert	130 / 143	0.91 (0.85 to 0.95)	5.6
Yanay	144 / 160	0.90 (0.84 to 0.94)	5.8
Zitt	46 / 47	0.98 (0.86 to 1.00)	1.5
**Summary (*I*-square = 78.6%)**		**0.87 (0.83 to 0.90)**	
**PD**			
Agur	22 / 23	0.96 (0.75 to 0.99)	21.9
Duarte	25 / 25	0.98 (0.76 to 1.00)	12.4
Longlune	17 / 20	0.85 (0.62 to 0.95)	43.6
Rodríguez-Espinosa	31 / 32	0.97 (0.81 to 1.00)	22.1
**Summary (*I*-square = 18.2%)**		**0.94 (0.84 to 0.98)**	

***P* for subgroup difference = 0.153**

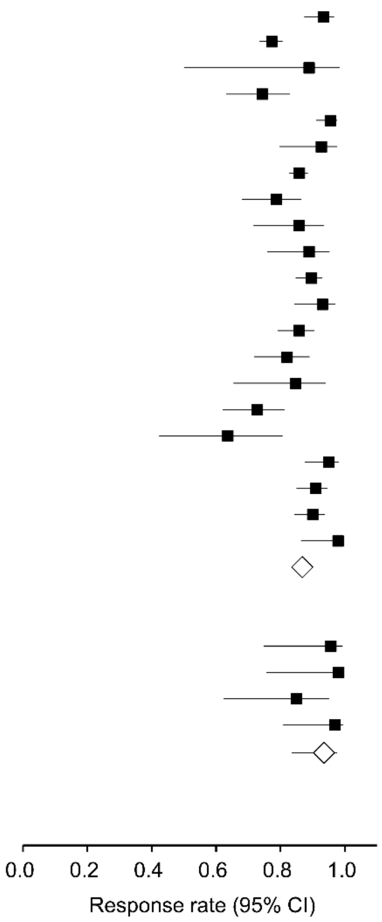

© 2021 Chen JJ et al. *JAMA Network Open.*

eFigure 4. Associations Between Age and Immunogenicity Rates in SARS-CoV-2–Naive Dialysis Population with Complete Vaccine Protocol

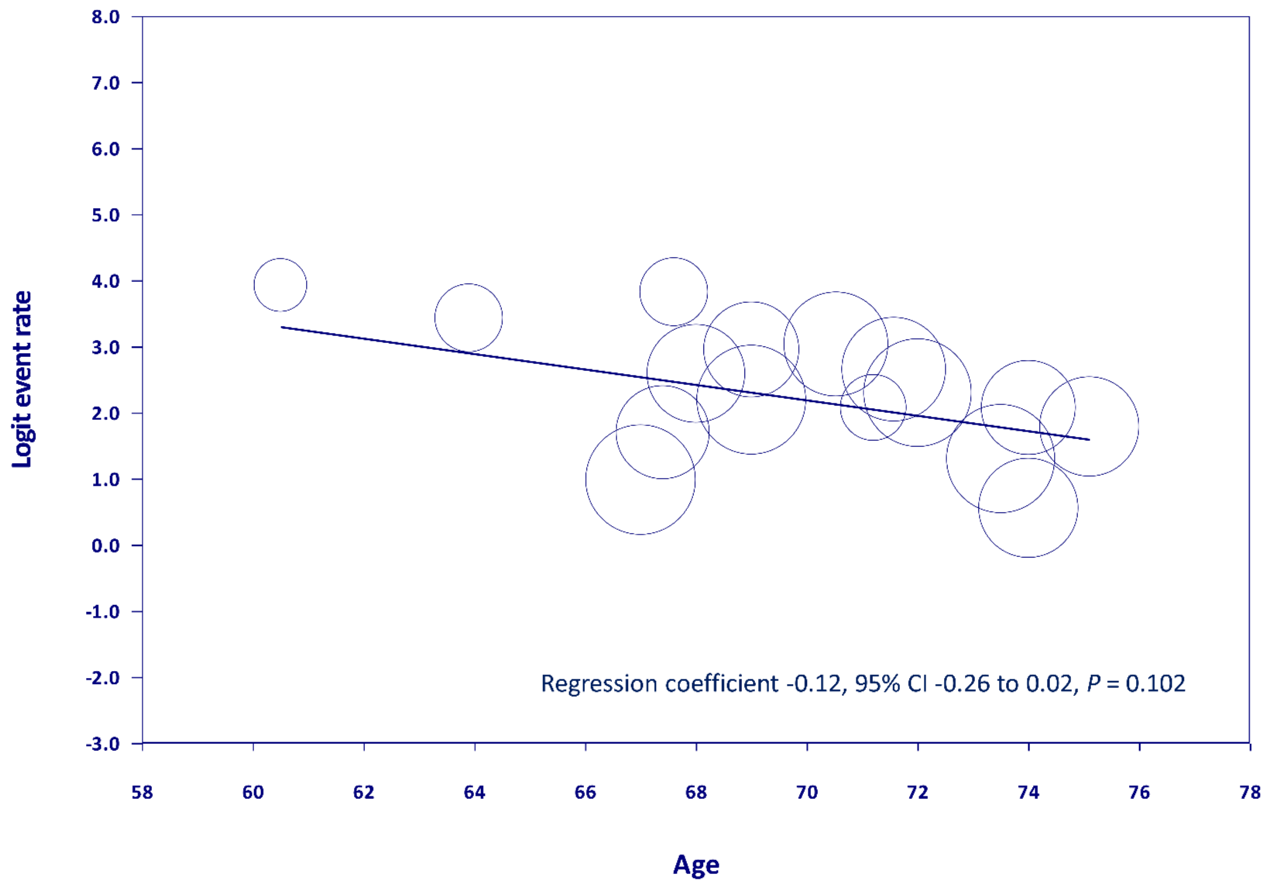

© 2021 Chen JJ et al. *JAMA Network Open.*

eFigure 5. Associations Between Proportion of Women and Immunogenicity Rates in SARS-CoV-2–Naive Dialysis Population With Complete Vaccine Protocol

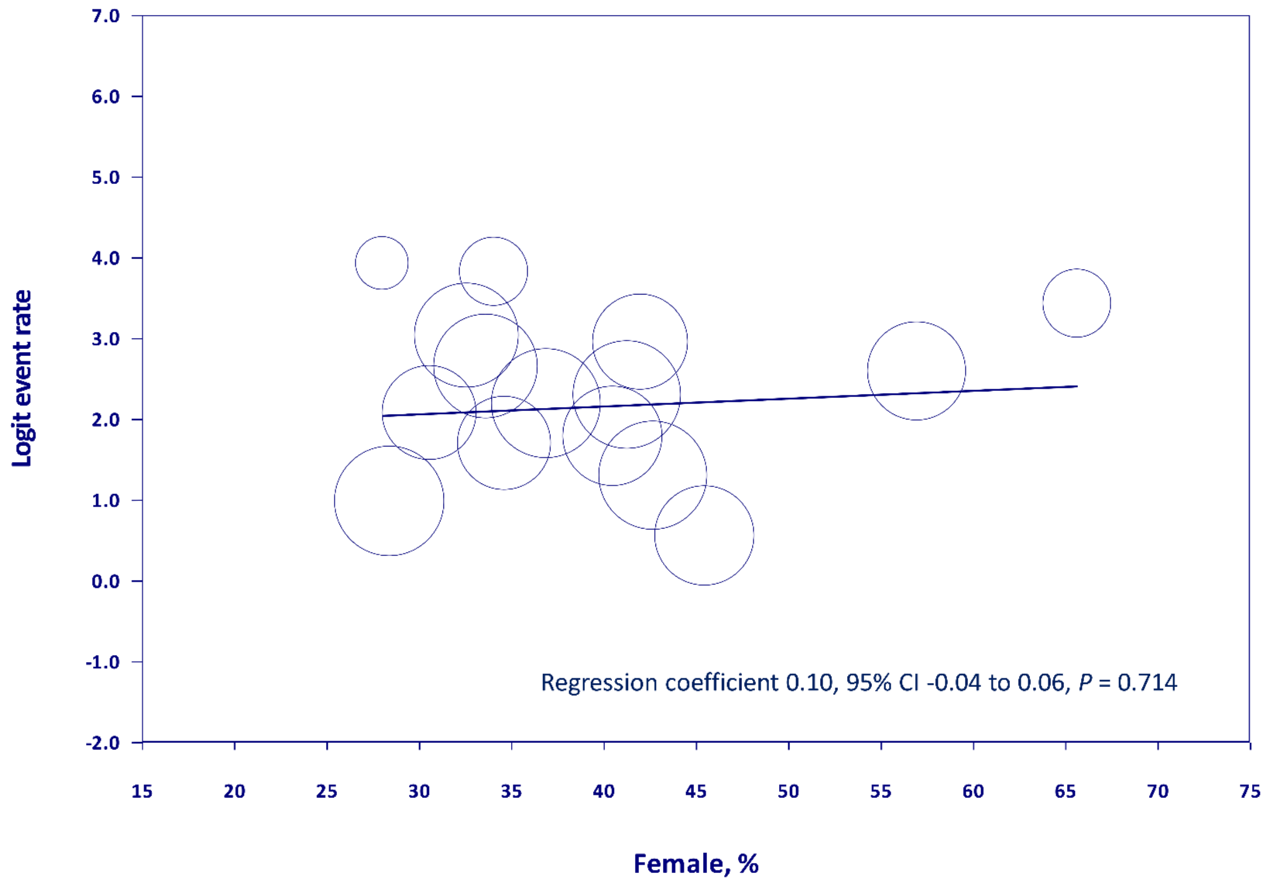

© 2021 Chen JJ et al. *JAMA Network Open.*

eFigure 6. Associations Between Dialysis Vintage and Immunogenicity Rates in SARS-CoV-2–Naive Dialysis Population With Complete Vaccine Protocol

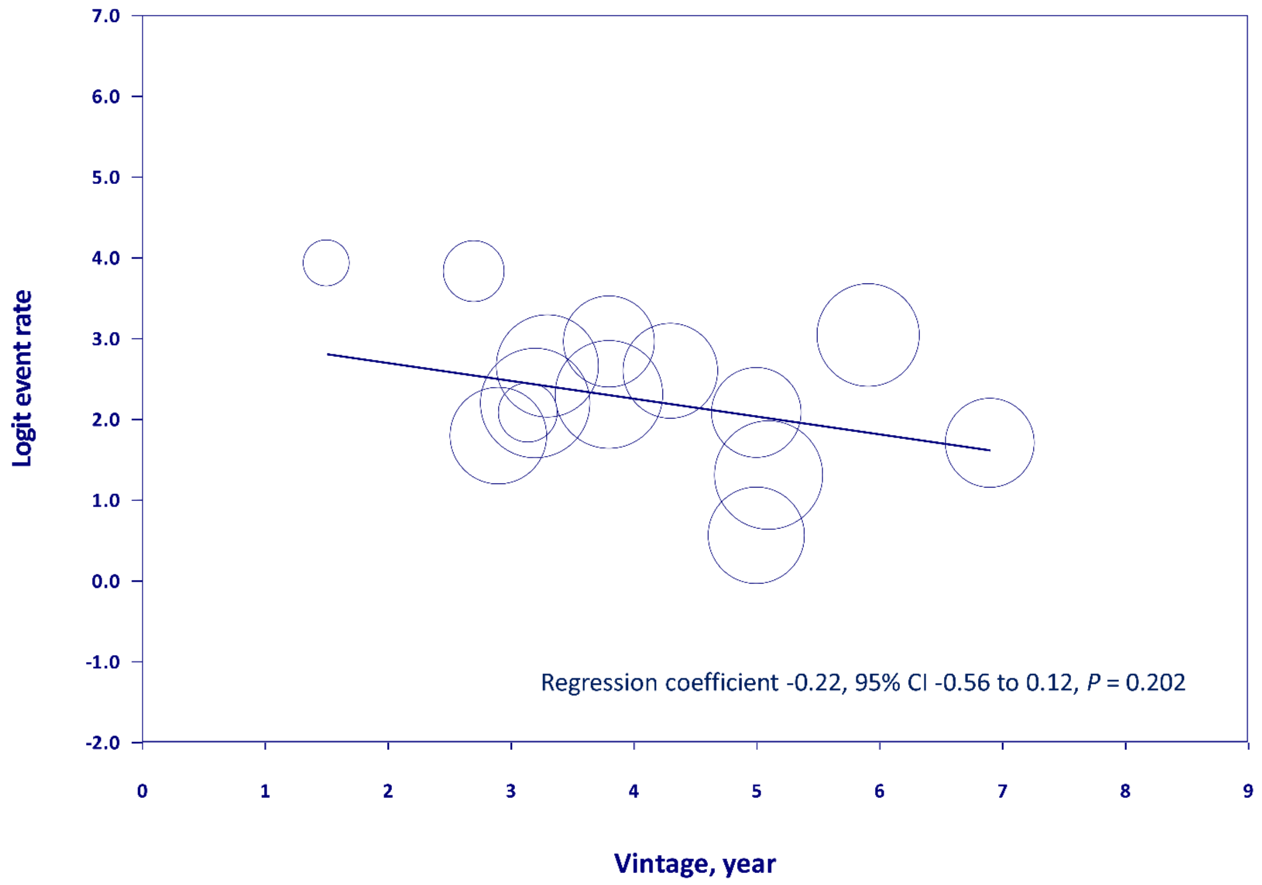

© 2021 Chen JJ et al. *JAMA Network Open.*

eFigure 7. Funnel Plot Illustrating Publication Bias

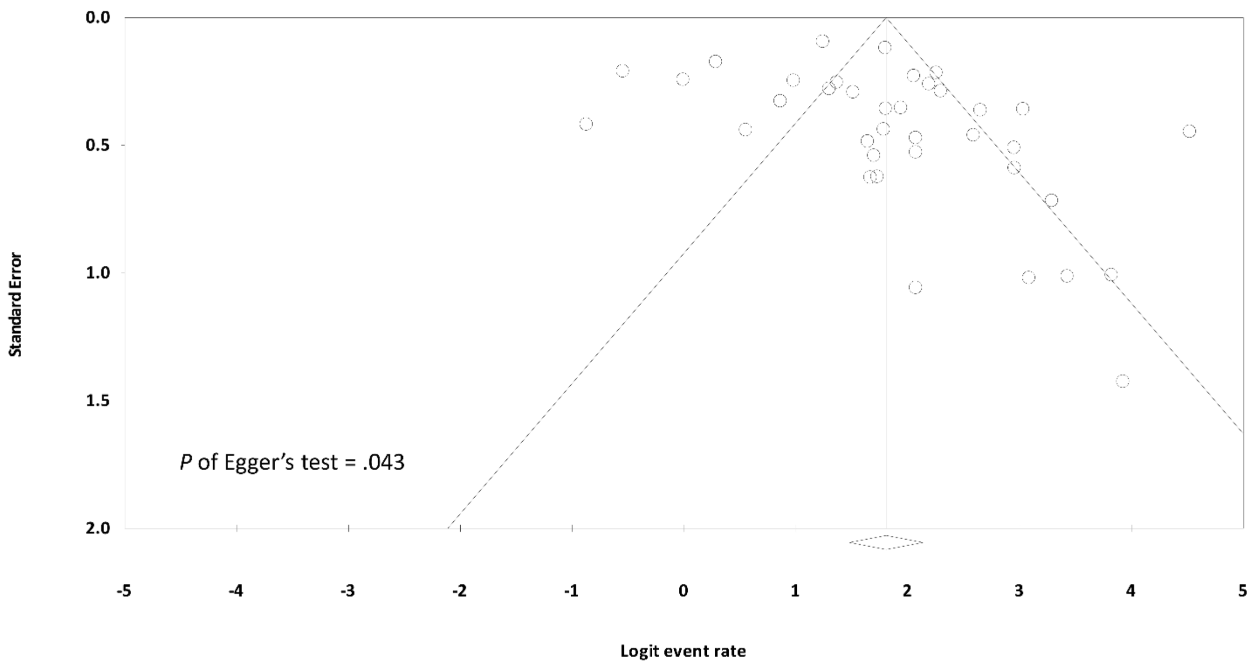

© 2021 Chen JJ et al. *JAMA Network Open.*
